# Supplementary material for: Titration of Androgen Signaling: How Basic Studies Have Informed Clinical Trials Using High-Dose Testosterone Therapy in Castrate-Resistant Prostate Cancer
Source: Life (Basel). 2021 Aug 27;11(9):884. doi: 10.3390/life11090884 (PMC8465783; doi:10.3390/life11090884)
Supplement: Supplementary file 1 [file life-11-00884-s001.zip › life-1346725-supplementary.pdf]

life

MDPI

Review

Titration of Androgen Signaling: How Basic Studies Have informed Clinical Trials Using High-Dose Testosterone Therapy in Castrate-Resistant Prostate Cancer

Steven K. Nordeen [1,†], Lih-Jen Su [2,†], Gregory A. Osborne [2], Perry M. Hayman [2], David J. Orlicky [1], Veronica M. Wessells [2], Adrie van Bokhoven [1] and Thomas W. Flaig [2,*]

[1] Department of Pathology, University of Colorado Denver Anschutz Medical Campus, Aurora, CO 80045, USA
[2] Division of Medical Oncology, Department of Medicine, University of Colorado Denver Anschutz Medical Campus, Aurora, CO 80045, USA
† These authors contributed equally to this work
* Correspondence: Thomas.Flaig@cuanschutz.edu

Supplementary Materials:

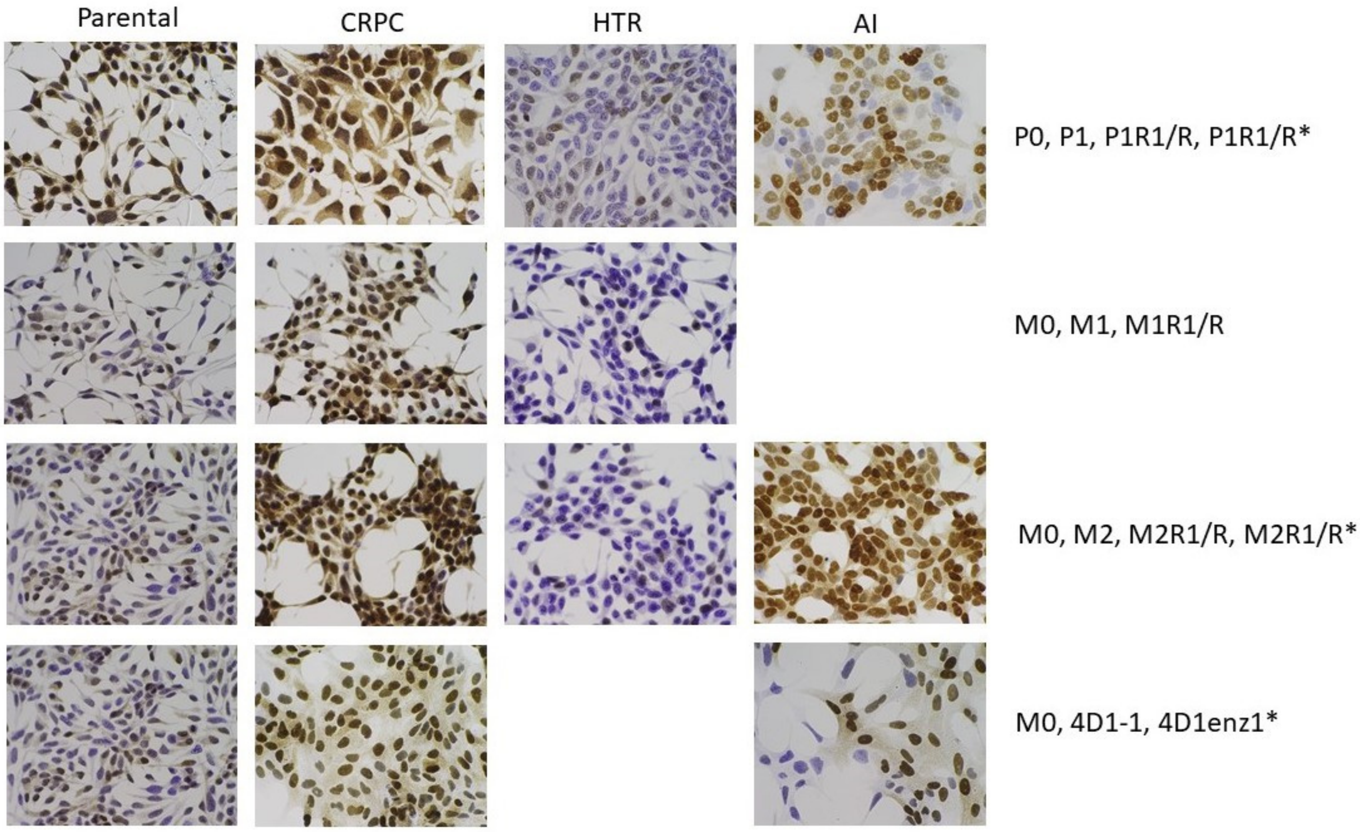

Figure S1. IHC for AR in variants of the different series described in Table one. Heterogeneity of AR expression is observed in parental lines grown in the modest androgen levels in medium supplemented with 10% fetal bovine serum. Variants selected by survival and growth medium supplemented with 5% charcoal-stripped fetal bovine serum represent models of CRPC. These variants all express elevated expression of AR. HTR variants were selected from the CRPC by survival and growth in the same medium supplemented with 1 nM R1881. They display reduced AR expression but more heterogeneity of expression levels than the CRPC lines from which they were derived. Variants that were capable of rapid adaptation to a second round of androgen withdrawal (AI-variants) were selected from HTR variants by removing the R1881-supplementation. These variants display substantial heterogeneity of AR expression with many cells expressing little or no AR despite the lack of androgen in the growth medium in contrast to the CRPC variants obtained in the first round of androgen deprivation.

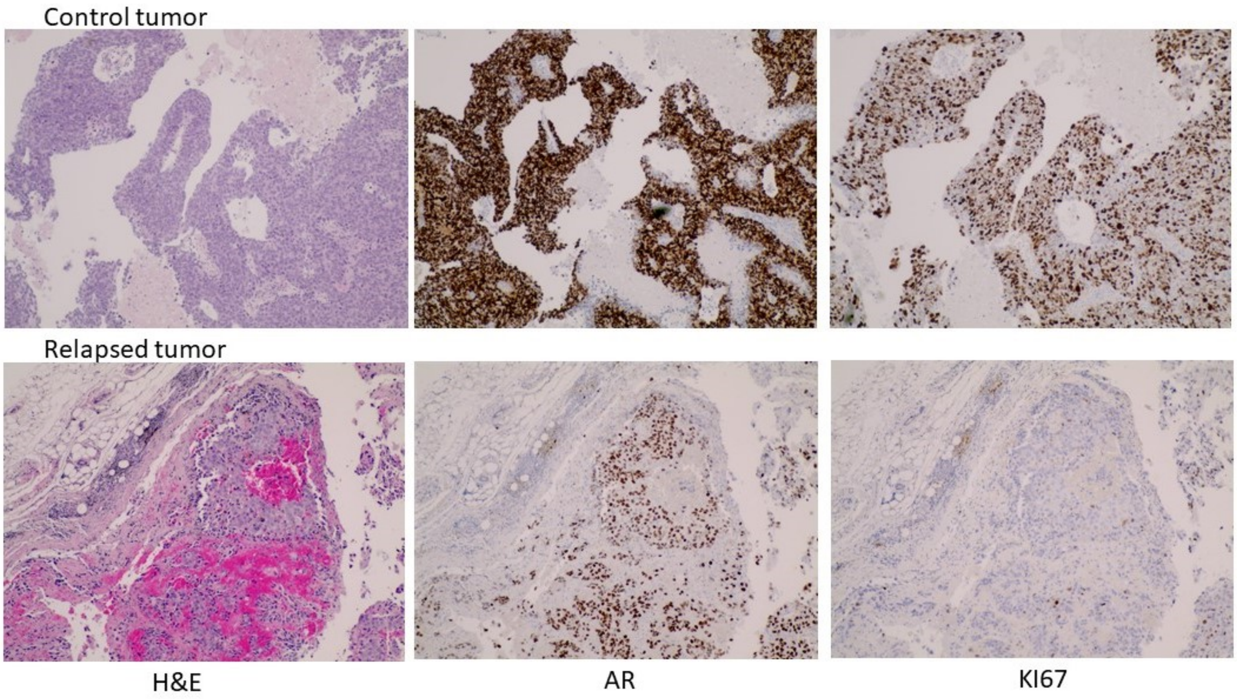

Figure S2. Wide field presentation of the slides in Figure 5. 40× magnification.

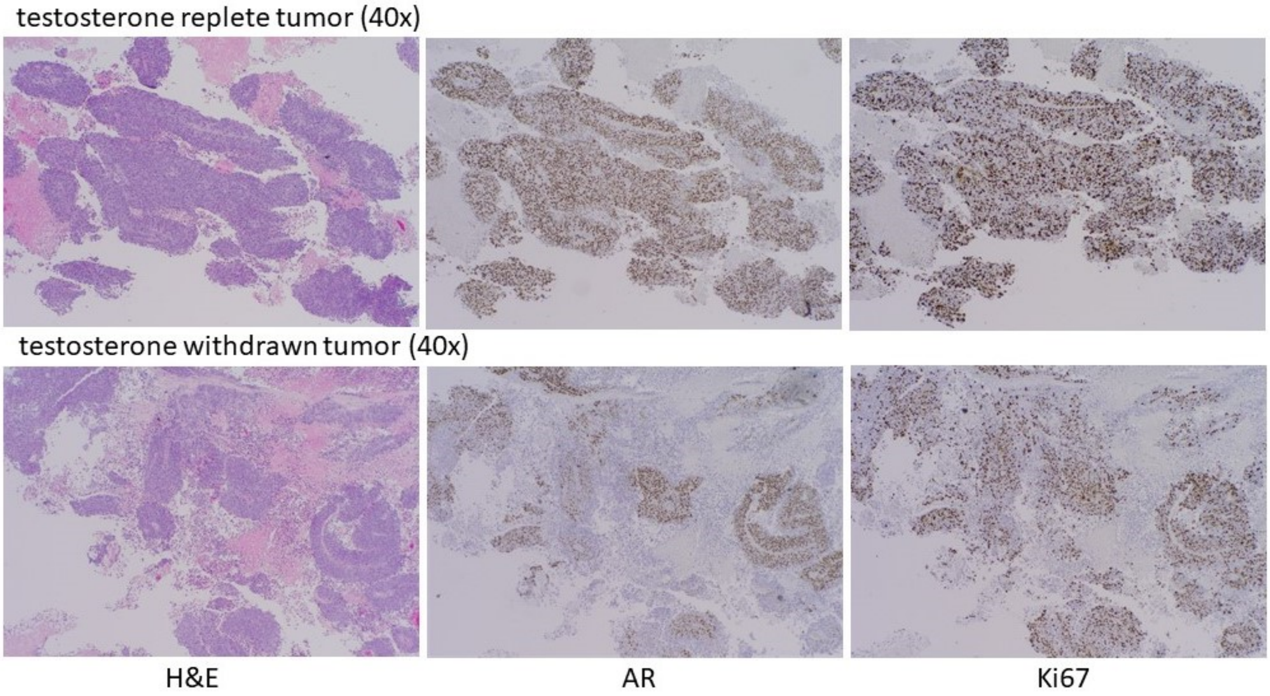

Figure S3. Wide field presentation of the slides in Figure 7. 40× magnification.

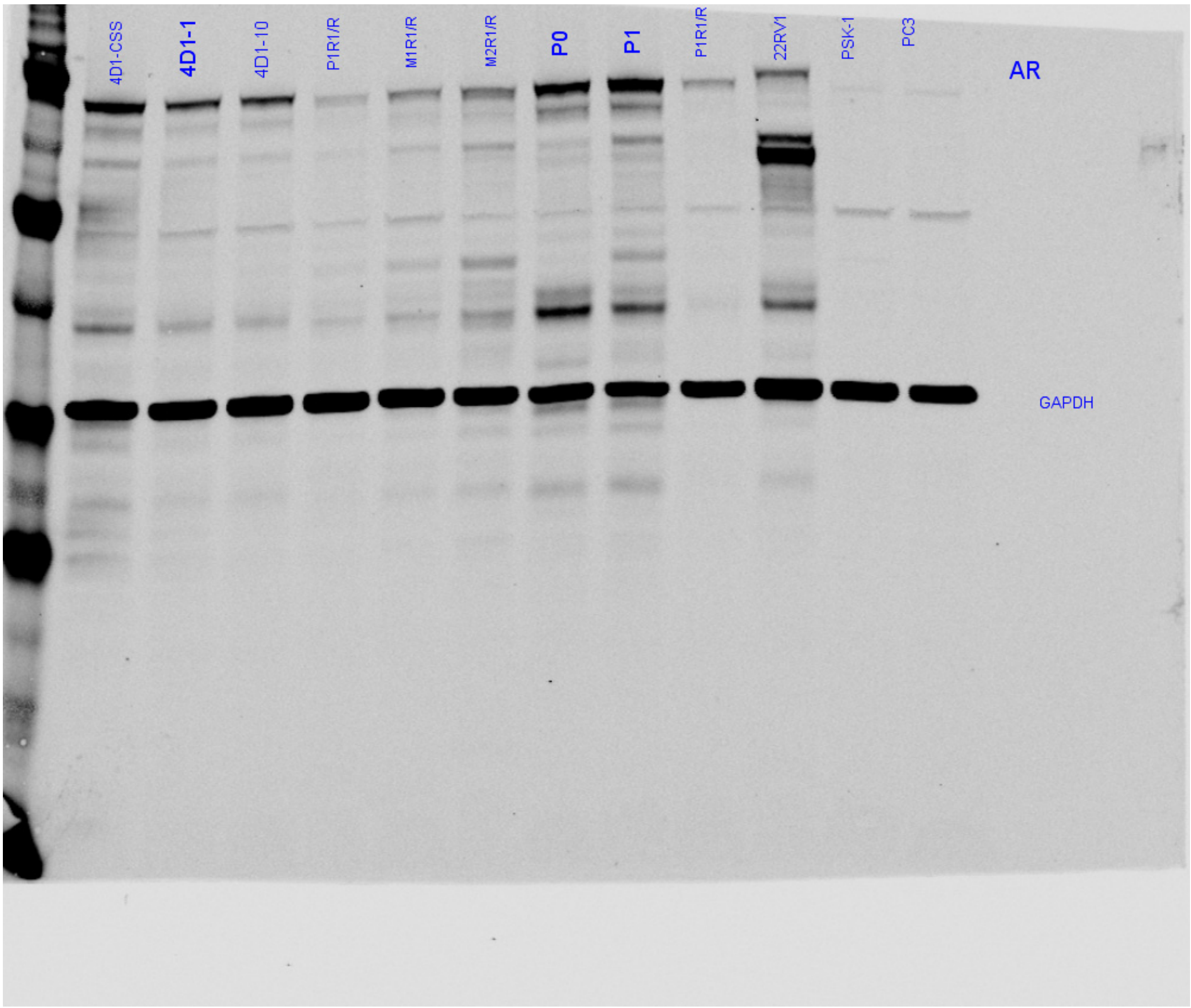

Figure S4. The entire Western blot from with the relevant data for AR and GAPDH have been extracted and presented in Figure 2A of the manuscript.
